# Progress in Self-Repair Technology for Concrete Cracks via Biomineralization

**DOI:** 10.3390/ma18215004

**Published:** 2025-11-01

**Authors:** Meirong Zong, Wenhao Wang, Haozhe Ma, Nshuti Cedrick, Yuting Sun, Xiancui Yan, Hui Liu, Pinghua Zhu, Minqi Hua

**Affiliations:** 1 Department of Civil Engineering, Changzhou University, Changzhou 213164, China; 19501141529@163.com (W.W.); 13525413493@163.com (H.M.); kencedrick54@gmail.com (N.C.); s24210814216@smail.cczu.edu.cn (Y.S.); yanxc@cczu.edu.cn (X.Y.);; 2 School of Civil Engineering & Architecture, Wuhan University of Technology, Wuhan 430070, China

**Keywords:** self-healing cracks, concrete bioremediation, enzyme-mediated processes, microencapsulation, calcium carbonate carbonation

## Abstract

Biomineralized self-healing concrete is a type of concrete that, during its service life, induces the generation of calcium carbonate through the participation of microorganisms or active enzymes, thereby achieving self-repair of cracks at different times. Self-healing concrete based on biomineralization can achieve sustainable crack repair and could enhance the strength and extend the service life of buildings. This article comprehensively analyzes the latest progress in bio-self-healing concrete, including microbial-based self-healing, enzyme-induced calcium carbonate precipitation (EICP), microcapsule-loaded microbial in situ remediation, and bio-inorganic mineral synergist self-healing technology. The maximum repairable width of the crack is 2.0 mm, and concrete strength can be increased by 135%. These methods offer new insights and strategies for the repair of concrete cracks, providing fundamental knowledge for the later application of intelligent engineering of bio-self-healing concrete and the analysis of micro-interface mechanisms. At the same time, they clarify the practical possibility of microbial technology in building materials science and engineering and offer key theoretical support for the long-term development of China’s construction industry.

## 1. Introduction

Concrete is one of the most widely used building materials in the world, with an annual output exceeding 60 trillion cubic meters [1,2]. However, concrete structures are susceptible to cracking due to excessive tensile stress or environmental erosion, which compromises their load-bearing capacity, increases the risk of structural instability, and may lead to catastrophic failures under high loads or extreme weather conditions [3,4]. Cracks in concrete structures can be primarily categorized into the following types: Load-induced cracks result from static, dynamic, or secondary stresses and are classified into direct stress cracks and secondary stress cracks [5]. Temperature-induced cracks arise from restrained thermal expansion or contraction due to internal or external temperature variations. Shrinkage cracks, the most prevalent type, include plastic, drying, autogenous, and carbonation shrinkage [6]. Frost-heavy cracks are caused by the expansion pressure and osmotic pressure generated when water within concrete freezes. Settlement cracks form due to additional stresses induced by uneven foundation settlement. Additionally, construction-induced cracks occur as a result of improper construction techniques or poor workmanship, particularly common in slender and thin-walled structural elements [7,8]. The deterioration of concrete infrastructure poses significant socioeconomic and environmental issues, as maintenance and rehabilitation of aging structures require 30% to 50% of overall construction spending [9,10,11,12]. As a result, identifying solutions to avoid fracture initiation, reduce maintenance costs, and mitigate the lifetime implications of repair treatments remains a critical concern for sustainable infrastructure development.

Traditional concrete repair strategies are typically passive, as illustrated in Table 1. Prior to any repair work, it is essential to conduct preliminary inspections to locate and assess cracks, with repairs generally limited to accessible and visible cracks [13]. Commonly used adhesives in coating-based repair methods include epoxy resins, polyesters, and vinyl esters [14]. However, these materials may form harmful surface films and emit toxic volatile substances, posing risks to both human health and the environment [15]. The filling method operates by injecting grouting materials into the crack cavity under relatively high pressure over a specific time period [13]. The secondary surface pressing method is applicable only to surface cracks and requires protection from dry conditions or direct sunlight to prevent premature cracking of the repaired cement. The surface tiling method is suitable only for minor cracks [16]. Secondary surfacing can only address surface-level fractures and must be avoided in dry settings or in direct sunlight to prevent early breaking of the repair material. Only fine cracks can be repaired using the surface patching approach. These methods are not only time-consuming and labor-intensive but also ineffective in addressing internal cracks in concrete structures. Therefore, it is urgently necessary to explore green and sustainable technologies and achieve simultaneous repair of internal and surface cracks.

**Figure 1:**
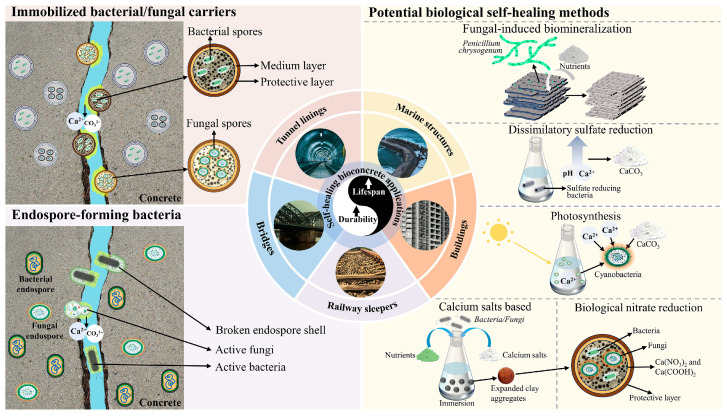
Overview of microbial mechanisms facilitating self-healing in concrete [24].

To overcome the limitations of standard concrete crack repair procedures, researchers worldwide have begun developing self-healing concrete technologies, allowing concrete to have self-monitoring and self-repairing capabilities [17]. Among these self-healing approaches, microbial-based concrete has demonstrated the most promising results due to its sustained effectiveness [18,19,20]. Microorganisms in concrete usually exist in the form of dormant bodies and remain active. Common forms of microbial dormancy include bacterial dormancy bodies, thick-walled fungal dormancy bodies and sclerotia. When the environment is suitable, dormant forms can resume life activities through germination mechanisms, such as the new development of bacterial dormant bodies into vegetative cells [21]. Water into freshly developed cracks activates latent microorganisms, causing them to proliferate and precipitate calcite (CaCO_3_), eventually healing the cracks. Once the cracks are sealed, the bacteria become dormant. If more fractures develop in the future, the microbes are reactivated to fill them. As a result, these microbes act as long-term healing agents, a process known as Microbially Induced Carbonate Precipitation (MICP), as illustrated in Figure 1 left part [2,11,22,23,24]. *S. pasteurii* (encapsulated), *Bacillus aerius*, *B. pasteurii*, *Bacillus*, etc., have all been shown through laboratory studies to be able to produce calcium carbonate precipitation under appropriate pH conditions. Subtilis the University of Technology of Delft (The Netherlands) development of bacteria-based self-healing concrete, a bacteria-based repair mortar and liquid system were developed for the treatment of aged concrete structures. Field trials have been carried out with either type of bacteria-based systems and the promising results have led to a spinoff company Basilisk Self-Healing Concrete with the aim to further develop these systems and bring them to the market, as illustrated in Figure 1 middle part [12,25]. Biological enzymes are also the main approach for microbial concrete repair. Urease is a bacterial metabolic enzyme that can catalyze urea, carbonate, and ammonium in the bacterial culture medium, thus increasing the carbon concentration and pH value [26], Promote the rapid formation of calcium carbonate to achieve crack repair as illustrated in Figure 1 right part.

This paper reviews the research progress in the healing mechanism of self-healing concrete cracks, microscopic analysis techniques, changes in concrete strength, and the advantages and disadvantages of engineering applications from aspects such as fungi, bacteria, mixed strains, enzyme-mediated, biological microcapsules, algae, and organic-combined inorganic microorganisms. Filamentous fungi in fungal technology, due to their excellent cell wall binding ability and metal absorption capacity, can increase the precipitation rate of CaCO_3_, making it more effective in the biomineralization process. The number of nucleation sites (such as cell walls) has a significant impact on the amount of precipitated minerals. Bacterial technology is a form of self-healing and is often used in self-healing concrete. This process can be achieved by converting lactic acid into calcium carbonate (CaCO_3_) precipitation or by using urea hydrolysis as a catalyst to promote the formation of CaCO_3_ precipitation [27]. Mixing technology is typically composed of multiple interacting microbial communities, which exhibit greater resistance to environmental impacts, enhance their survival capabilities under various conditions, and adapt to different factors in concrete, such as changes in pH value, temperature, and moisture content [28]. The plant urease was extracted by enzyme-mediated method for biological cementation to repair cracks in concrete structures. The response is rapid and direct, highly efficient, and the microbial culture process is omitted. Therefore, using plant-based urease for biological cementing to repair cracks in concrete structures is a cost-effective and environmentally friendly alternative [29]. Algal restoration is a mineralization process driven by photosynthesis, mild, environmentally friendly and precisely regulated by organic substrates. It demonstrates unique and huge potential in macro-ecological shaping, carbon sequestration and restoration in specific fields [30]. Microencapsulation Technology: When a crack forms, the microcapsules within the cement-based material may rupture, releasing the repair agent contained in the microcapsules into the cracks. This agent then interacts with the catalyst, enabling the cracks to self-heal. While this repair process is highly effective, the repair agent is primarily organic, challenging to package, difficult to transport to the damaged location after release, has a short service life, and negatively affects the material’s strength [31]. This review provides a reference for the self-repair of cracks in concrete structures and the extension of their service life in the future.

## 2. Advances in Biological Self-Healing Research

### 2.1. Repair Methods of Fungi, Bacteria and Biological Communities

*Fungal Remediation Techniques*

Filamentous fungi exhibit exceptional tolerance to harsh environments, thriving at temperatures ranging from −10 °C to 40 °C [24] and pH levels of 7.5–11.0 [32]. Many fungi produce organic acids such as citric acid and gluconic acid during their metabolic processes. These organic acids can initially dissolve calcium-containing substances such as calcium hydroxide (Ca(OH)_2_) in concrete, releasing more Ca^2+^. As the reaction proceeds, organic acids are consumed or ammonia gas is produced, causing the pH value of the environment to rise. At this point, the high concentrations of Ca^2+^ and CO_3_^2−^ (from air or metabolism) in the solution reach supersaturation, thereby forming calcium carbonate precipitate.

**Figure 2:**
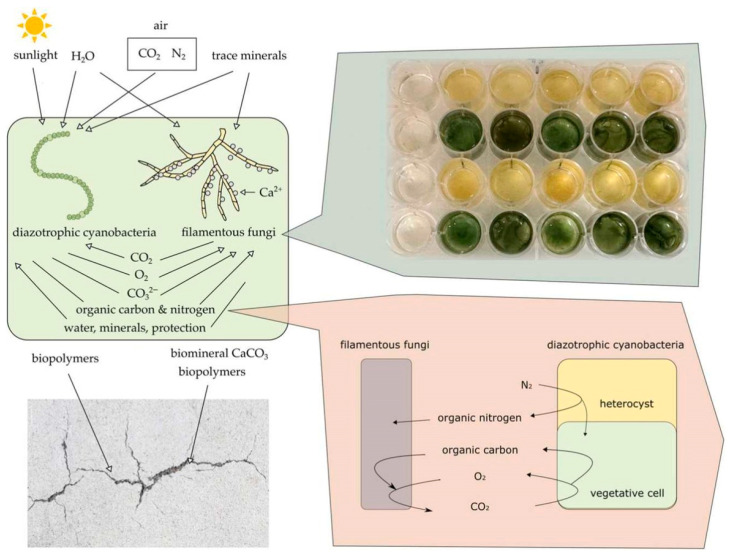
Sheshidu filamentous fungi repair concrete by absorbing nitrogen and carbon dioxide from the air (temperature 25 to 30 °C, pH 12) [34].

Khushnood et al. [33] demonstrated that Rhizopus oryzae and Trichoderma longibrachiatum adsorb Ca^2+^ and induce CaCO_3_ precipitation via urea hydrolysis, with mycelium-CaCO_3_ composites effectively healing cracks up to 1.3 mm (Figure 2). Luo and Chen [34] reported Trichoderma reesei germination and growth in pH 11.0 concrete. Zhao et al. [35] Isolated Fusarium and Mucor strains from concrete that produce ammonia nitrogen and precipitate calcite through urea hydrolysis at pH 8.3–11.0. Zhang and Fan [36] found Fusarium oxysporum healing efficiency varied with pH, requiring 11.3–79.6% of MICP healing time. Khan et al. [37] showed Fusarium oxysporum and Trichoderma longibrachiatum increased concrete compressive/tensile strength by 11.62% and 31.18% after 28 days, reduced water absorption by 0.8%, improved acid resistance by 4.5%, and completely sealed 1.34 mm cracks, restoring 58.75% of compressive strength. Filamentous fungi have a high remediation effect, mainly due to their branching structure and filamentous growth habits, which provide more nucleation sites and stronger skeletal support for mineral precipitation [38]. The extracellular polymers of fungi are rich in chitin and glycoproteins, which can adsorb a large amount of metal ions [39], Through urea hydrolysis and organic compound conversion processes and absorb CO_2_ from the air and complete microbial induced calcium carbonate precipitation (MICP) to achieve crack repair [40]. In urea hydrolysis, urease degrades urea into HCO_3_^−^ and NH_4_^+^, increasing pH and promoting CO_3_^2−^ formation, which reacts with Ca^2+^ to precipitate CaCO_3_.

*Bacterial Remediation Technology*

**Figure 3:**
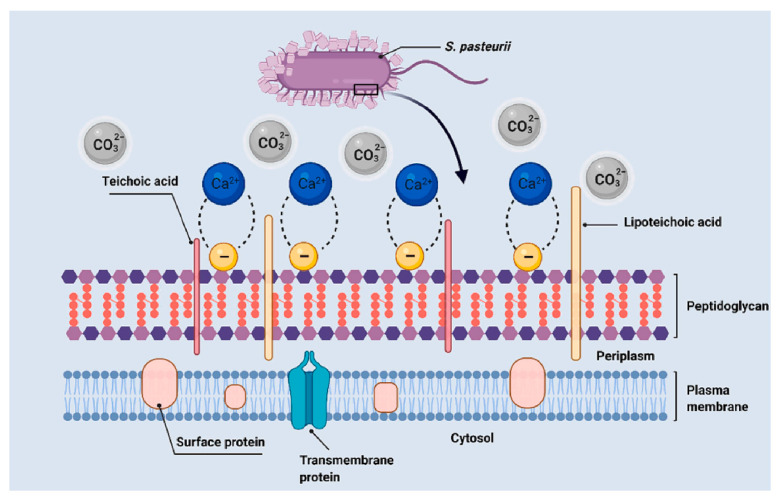
Schematic diagram of the nucleation site and reaction mechanism of bacterial cell walls (Taking Vibrio pasteurella as an example, pH 12.5, temperature 25 °C) [42].

In the process of biomineralization, carbonate minerals produced by bacterial metabolism are the main source of concrete crack repair materials [41]. Yang et al. [42] reported the self-healing mechanism of concrete cracks under bacterial action, which mainly consists of two steps: The first step is oxygen and water penetrate into the concrete through the cracks, activating the bacteria in the cracks, which convert calcium lactate into calcium carbonate crystals and carbon dioxide. Calcium carbonate crystals can repair the internal cracks of the concrete. Concrete particles near the cracks will further react with carbon dioxide to generate more calcium carbonate, which precipitates on the crack surface, thereby repairing the concrete surface cracks. In the second step, many components capable of producing organic urea can act as catalysts in the self-healing process. During demineralization [43], negatively charged bacterial cells absorb Ca^2+^ ions through the cell wall and then react with CO_3_^2−^ precipitates (Figure 3). Currently, microbial-induced calcite precipitation (MICP) technology based on urea hydrolysis dominates engineering applications due to its high efficiency and excellent controllability. Both cells and extracellular polymers can serve as nucleation sites, and the negatively charged properties of cell walls and extracellular polymers exert electrostatic attraction on calcium ions. This provides a useful basis for further understanding and identifying biogenically induced carbonate deposits. The type of microorganism directly affects the repair efficiency of concrete. Chen and Jun [9] cultured alkali-resistant *Bacillus* subtilis M9, using air-entraining agents to create micropores in cement paste mixtures, providing ecological niches for microorganisms. After urea hydrolysis, microcracks achieve autonomous healing through calcium carbonate precipitate filling. In addition, the flexural strength of sealed beams subjected to repeated bending increased by approximately 14%.

Durga [41] et al. found that when B. subtilis with a cell concentration of 10^8^ cells/mL was incorporated into concrete, the splitting tensile strength increased by 16% after 28 days of crack curing. Similarly, the addition of B. sphaericus increased the splitting tensile strength of concrete by 13.75%, 14.28%, and 18.35%, respectively. Sandip Mondal et al. [42] used three different concentrations of *Bacillus* subtilis: 10^3^ cells/mL, 10^5^ cells/mL, and 10^7^ cells/mL. The results showed that the crack healing efficiency was higher when the bacterial concentration was 10^7^ cells/mL, but the enhancement effect on crack compressive strength was optimal when the bacterial concentration was 10^5^ cells/mL. Zhang and Weng [44] studied the method of transposon mutagenesis to modify the genes of halotolerant *Bacillus* by catalyzing the combination of carbonate and calcium ions, obtaining mutant strains with higher calcium carbonate production efficiency. Bacterial concentration significantly influences the polymorphic forms of calcium carbonate (CaCO_3_) [45].

Calcium carbonate can exist in three crystalline polymorphs: calcite, vaterite, and aragonite. Among these, calcite is the most thermodynamically stable phase owing to its low solubility and high structural integrity. Fracture specimens precipitated under lower bacterial concentrations exhibit enhanced cementation characteristics, which offers a microstructural rationale for the observed reduction in dynamic strength in specimens treated with higher bacterial concentrations. Therefore, the optimal bacterial concentration range for effective crack healing lies between 10^5^ and 10^7^ cells/mL [46].

Above all, fungal repair of concrete cracks is currently more in the exploratory research stage [47]. It may have certain value as an auxiliary means in specific scenarios (such as surface coating, combined with porous lightweight materials), but its inherent biological characteristics (such as acid production, slow growth, and mainly physical filling) make it difficult to compete with mature bacterial mineralization repair technologies in terms of efficiency, universality, long-term stability, and compatibility with substrate materials at present. Bacteria-induced calcium carbonate deposition remains the most mainstream and promising technical approach in bio self-healing concrete. During the reaction process, calcium carbonate crystals chemically combine with the concrete matrix, effectively restoring mechanical properties. The strength recovery rate usually reaches over 90% (Table 2).

*Mixed Bacteria Remediation Technology*

**Figure 4:**
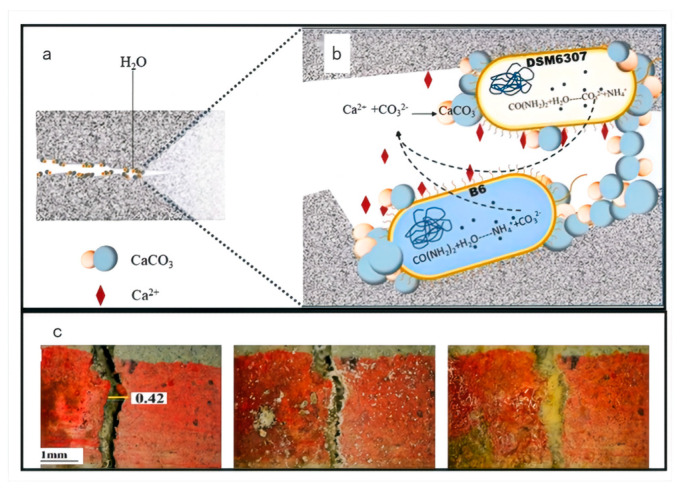
(**a**) Self-healing effect of mixed bacteria (DSM6307, B6) with starch and ammonium nitrate on concrete CaCO_3_; (**b**) precipitation Concrete healing effect drawing, (**c**) A physical picture of the healed crack [43].

Microbial communities, also known as mixed cultures, typically comprise multiple interacting microbial populations. These communities demonstrate enhanced resistance to environmental fluctuations, improved survival under diverse environmental conditions, and greater adaptability to dynamic concrete environments, including variations in pH, temperature, and moisture content [48]. The application of mixed cultures for different crack types can reduce dependency on single bacterial strains and lower overall repair costs. An, Xu et al. [49] explored the feasibility of enhancing recycled concrete aggregates (RCAs) through the use of microbial consortia. Their experimental findings revealed that the physical and mechanical properties of RCAs improved significantly with extended biological deposition periods. Huang and Du [50] investigated the repair of concrete cracks using a multi-bacterial system consisting of *Bacillus* megaterium and Saccharomyces cerevisiae, assessing repair efficacy through crack healing observation, permeability testing, acoustic time-of-flight measurement, and unconfined compressive strength analysis. Results demonstrated that a microbial composition ratio of 30% Saccharomyces cerevisiae to 70% *Bacillus* megaterium yielded a 10% increase in calcium carbonate production, achieving optimal mineralization and deposition efficiency. Muhammad Arslan Ahmad and Zhang [51] focused on developing self-healing concrete by leveraging the synergistic mechanisms of mixed bacterial strains (Figure 4). When strains B6 and DSM6307 were applied at an initial concentration ratio of 4:6, CaCO_3_ precipitation was significantly enhanced. Nutrient sources were found to play a critical role in modulating bacterial interactions, with starch and ammonium nitrate identified as the most effective carbon and nitrogen sources, respectively. SEM/EDS (Scanning Electron Microscopy/Energy Dispersive Spectroscopy) analysis confirmed the formation of thicker calcite layers in co-cultured systems, indicating that bacteria-induced mineralization effectively sealed micropores and microcracks. The utilization of mixed cultures thus offers a promising strategy to minimize reliance on individual strains and reduce maintenance costs. A deeper understanding of these synergistic interactions provides a viable pathway to enhance the performance and durability of concrete in practical engineering applications [52].

### 2.2. Microbial Remediation Reaction Mechanism

**Figure 5:**
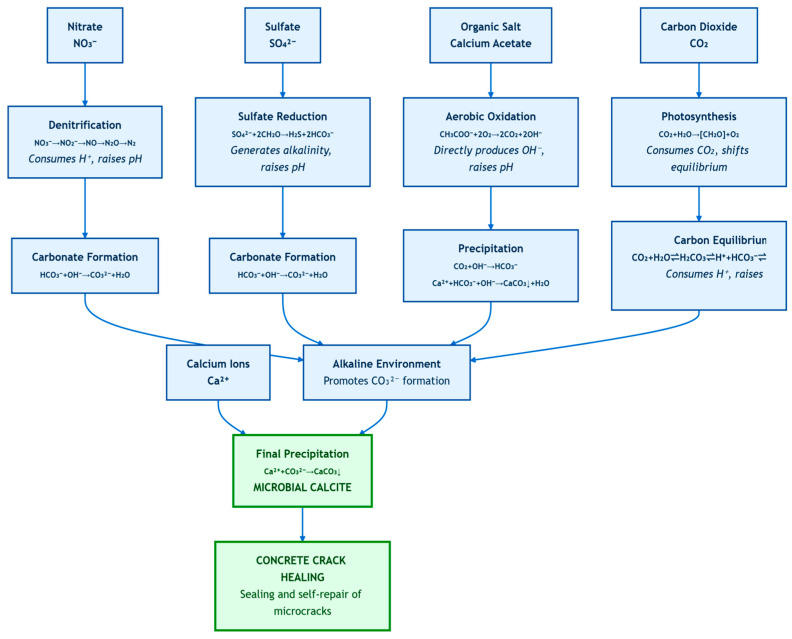
Specific equations for microbial reactions (nitrate reduction, sulfuric acid reduction, photosynthesis).

There are many differences in the self-healing mechanism of concrete microorganisms under different conditions. Such as nitrate reduction, sulfate reduction, oxidation of organic compounds and hydrolysis of carbon dioxide. Common reaction types include urea hydrolysis. Urea hydrolysis machine [53], nitrate reduction [54], sulfate reduction [55]. Oxidation of organic compounds and carbon dioxide hydrolysis [56]. The chemical reaction equation is shown in the following Figure 5.

Nitrate reduction, also known as denitrification, is the process of converting nitrate to nitrite, then to nitric oxide, nitrous oxide, and finally to nitrogen. Some anti-nitrifying bacteria enhance CaCO_3_ synthesis via this route. The denitrification (reduction) process is simplified in. This causes a rise in pH and the formation of carbonates. Finally, calcium interacts with carbonate to produce CaCO_3_.

The sulfur cycle can be utilized to reduce calcium sulfate, which can then be converted into CaCO_3_. This requires the presence of calcium, organic materials, and sulfates. The first step is to convert calcium sulfate into calcium sulfide. As a result of this process, the pH of the medium typically rises. This catalyzes the creation of CaCO_3_.

The heterotrophic growth of diverse microbes on various organic salts (e.g., acetate) results in the formation of carbonate minerals. In this process, numerous microbes consume organic salts to produce CaCO_3_.

In oxygenated photosynthesis, water works as a reactant, producing oxygen. Carbon dioxide is then extracted from the bicarbonate solution, yielding carbonate compounds. This causes a local pH rise, catalyzing the formation of CaCO_3_ in the presence of Ca^2+^.

### 2.3. Biological Enzyme Repair Technology

In recent years, Enzyme Induced Calcium Carbonate Precipitation (EICCP) technology has been widely used in the field of civil engineering. The representative study of EICCP is directly extracts urease from plants, catalyzes the hydrolysis of urea into carbonate ions, and reacts with calcium ions to produce calcium carbonate precipitation [57]. The principle is to introduce urease and reactive substances to generate calcium carbonate precipitates inside and on the surface of concrete, thereby filling the pores. Calcium carbonate can promote the healing of concrete cracks and improve the water resistance of cement-based materials. During urea hydrolysis, one molecule of urea is converted into one molecule of carbamate and one equivalent of ammonia (1) [58,59]. Then, the carbamate undergoes hydrolysis, producing another molecule of ammonia and one equivalent of carbonic acid (2). The carbonic acid formed in the previous step ionizes in water to form bicarbonate ions and hydronium ions (3). The ammonia produced in the urea hydrolysis and carbamate hydrolysis steps react with water to generate ammonium ions and hydroxide ions. Carbonate ions are formed when bicarbonate ions react with hydroxide ions. The negatively charged cell wall (4) attracts cations such as Ca^2+^ from the surrounding environment. These Ca^2+^ ions then interact with carbonate ions, leading to the formation and deposition of CaCO_3_ on the bacterial surface (5). This CaCO_3_ serves as a nucleation site, promoting further precipitation [60]. The generated free urease is biodegradable and will not have a long-term impact on the environment. Therefore, using plant-based urease for biological cementation in the repair of concrete structural cracks is a more economical and environmentally friendly method.

CO(NH_2_)_2_ + H_2_O → NH_2_COOH + NH_3_ (1)

NH_2_COOH + H_2_O → NH_3_ + H_2_CO_3_ (2)

2NH_3_ + 2H_2_O → 2NH^4+^ + 2OH^−^ (3)

2OH^−^ + H_2_CO_3_ → CO_3_^2−^ + 2H_2_O (4)

Cell-Ca^2+^ + CO_3_^2−^ → Cell-CaCO_3_ (5)

Long and Du [61,62] used soybean urease-induced calcium carbonate deposition to repair high-temperature damaged concrete. Studies found that soybean urease exhibits good activity at 40 °C to 70 °C and pH 7 to 8. The repair effect was optimal when the solution concentration was 1.0 mol/L, and the vacuum method with a negative pressure of 0.095 MPa showed better repair efficiency. Junwale, Rishabh D. and Bhutange, Snigdha P [63] directly added urease-containing watermelon seeds (in powder form) into mortar, which was identified as an effective bio-cementing agent in mortar, confirming that powdered seeds may help reduce the cost of bio-cementation. Junwale, Rishabh and Nikode Aishwarya [57] confirmed that the proposed bio-cementing suspension can completely repair cracks within 7 days after application. The repair process involves introducing a special suspension composed of urea, watermelon seed urease, and calcium hydroxide into existing cracks in specimens. Comparison before and after treatment showed that the compressive strength of cracked mortar specimens decreased by 35%, and the compressive strength recovered by approximately 90% on the 7th day.

**Figure 6:**
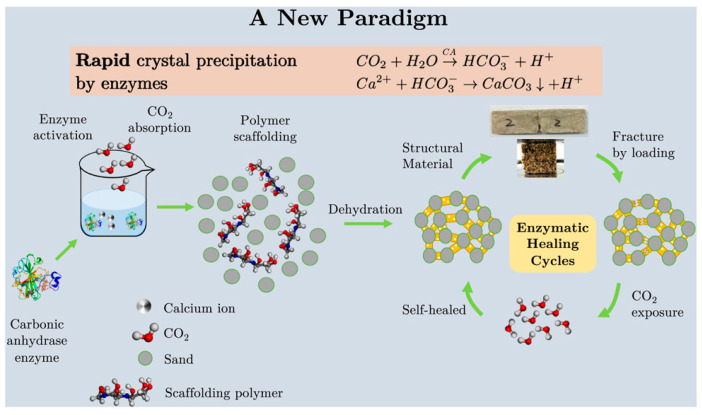
Carbonic anhydrase (CA) catalyzes the hydration of CO_2_ and promotes the precipitation of calcium carbonate (temperature 25 °C, pH 13, adapted from Ref. [67]).

With the continuous development of research, the enzymatic remediation technology of concrete is constantly innovating. Yang and Li [64] investigated the effects of two nucleating agents on EICP: adding sorbitol (optimal content of 5%) increased EICP yield by 5.1%, and adding sucrose (optimal content of 4%) increased EICP yield by 12.3%. After adding sorbitol (5.2%), the UCS (Unconfined Compressive Strength) of EICP-treated sand increased by 2.2 times, and the CaCO_3_ content of EICP-treated sand with sorbitol was 1.5% higher than that of conventional EICP-treated sand. Ding and Wang [65] studied two enzyme source solutions; seawater-acclimated *Bacillus* Pasteurian could better adapt to the high-salt environment of seawater, microbial metabolism was not inhibited, urea decomposition capacity was enhanced, and calcium carbonate production was higher. This can effectively improve the engineering properties of coastal sediments and play a positive role in coastal protection and development. Rosewitz and Wang [66] introduced a method to develop a self-healing mechanism in cementitious matrices using trace carbonic anhydrase (CA). CA catalyzes the reaction between Ca^2+^ ions and atmospheric carbon dioxide, forming calcium carbonate crystals with thermodynamic properties similar to the cementitious matrix. The crystal growth rate of this method is faster and more efficient than bacterial methods. Suzanne F. Scarlata and Wang [67] proposed using carbonic anhydrase (CA) to catalyze the condensation of CO_2_ with water, promoting the precipitation of calcium ions in aqueous solution in the form of calcium carbonate crystals (Figure 6), forming mineral bridges. The compressive strength and Young’s modulus of the resulting ECM (Engineered Cementitious Material) are more than twice the acceptable minimum values for cement mortar and other alternative building materials.

The most prominent advantage of enzyme repair technology is its repair speed, which can be completed within 24 h. Moreover, the repair product (calcium carbonate) has good compatibility with the concrete body and can effectively extend the service life of the structure by up to 80 years. At the same time, it has a cost advantage over the bacterial repair method. These characteristics make it have great potential in dealing with early micro-cracks in concrete. However, this technology is still mostly in the laboratory research stage at present, and there are few large-scale engineering application cases. Meanwhile, the CO_2_ and NH^4+^ generated during the remediation process will also impose a certain burden on the environment. In conclusion, it is one-sided to label EICP technology as completely “green” or “environmentally friendly”. While it offers outstanding engineering performance (crack repair, soil reinforcement), it does indeed come with the risks of nitrogen pollution and direct or indirect greenhouse gas emissions.

### 2.4. Microalgae Remediation Technology

Algal microbiologically induced carbonate precipitation demonstrates rapid growth kinetics and robust photosynthetic activity, offering significant advantages for the production of self-healing concrete. Researchers can harness the photosynthetic capacity of microalgae to facilitate crack repair while simultaneously sequestering carbon dioxide within the concrete matrix, effectively promoting the transformation of CO_2_ into calcium carbonate. This approach enables two critical functions: it enhances microbial carbonate precipitation for autonomous crack healing and contributes to carbon dioxide (CO_2_) capture, thereby reducing the carbon footprint associated with concrete production. This dual functionality holds promise not only for improving the structural resilience of concrete but also for enhancing its environmental sustainability [68]. Although research on microalgae-based self-healing concrete is still in its early stages, the preliminary results indicate considerable potential.

Rishiram Ramanan and Krishnamurthi Kannan [69] reported that at a CO_2_ input concentration of 10%, the carbon fixation efficiencies of *Chlorella* and *Spirulina* reached 46% and 39%, respectively, with both species producing calcite as a biomineralization product. The feasibility of algal carbon dioxide capture has thus been experimentally validated. Chandra, Subarna Bhattacharya et al. [70] conducted preliminary investigations into the characteristics of exopolysaccharides (EPS) secreted by cyanobacteria and microalgae. Their findings indicated that microalgae exhibited the highest effectiveness among algal species in promoting fracture healing. The evaluation of three algal species based on doubling time revealed that microalgae had the shortest replication periods. Additionally, the highest in vitro EPS production was observed in phosphorus-rich cultivation conditions. Fatma M. Taher [62,71] explored the partial replacement of cement with two concentrations of seaweed extract (30 and 50 mg/kg). Results showed that incorporating 50 mg/kg of seaweed extract increased the compressive strength of concrete by 42.55%, 9.13%, and 15.43% on the 3rd, 7th, and 28th days, respectively. Scanning electron microscopy revealed abundant calcite formation, which was chemically confirmed through EDX analysis. Panagiota D. Natsiopoulou et al. compared the CaCO_3_ generation rates of *Acutodesmus obliquus* under standard culture conditions and air-dried states. Air-dried *A. obliquus* cultures at 25 °C produced higher levels of carbonate precipitation than live cultures. At 70 °C, nucleation and crystal growth were inhibited due to thermal disruption of the microalgal molecular structure, whereas calcite precipitation in air-dried cultures resulted in a doubling of precipitation rates.

**Figure 7:**
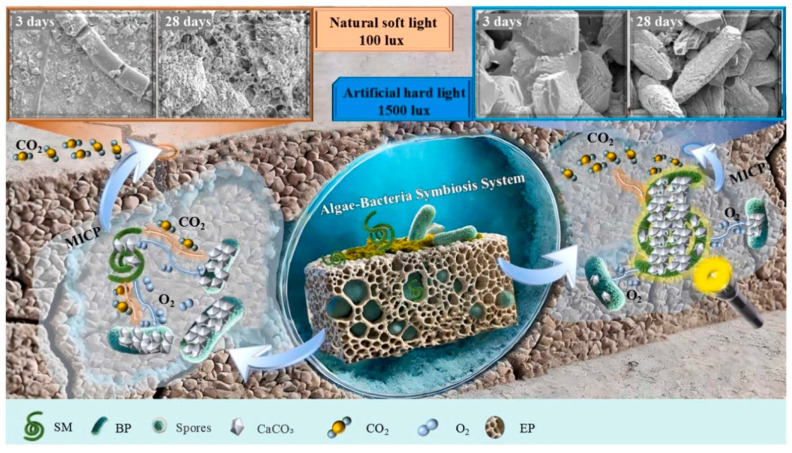
Schematic diagram of the reaction mechanism of algae repairing concrete cracks under 12 h of artificial light and 12 h of self-light [72].

Karthick Srinivas M and U. Johnson [72] utilized synchronous elongatus and spirulina platensis as micro-crack healing agents in cement mortar, replacing cement with 4%, 8%, and 12% spirulina. Their finding indicated that the combination of microalgae and cement exhibits a self-healing effect, demonstrating potential for enhancing the future fracture healing strategies (Figure 7). Additionally, Lee, Yi-Ying, and Jonas, Lauren [73] investigated that microalgae-driven processes for calcium carbonate and biomass production processes can efficiently capture and store atmospheric carbon dioxide in the form of calcium carbonate. This process results in calcium carbonate precipitation in high-pH, high-alkalinity microalgae cultures. Zhang and Liu [74] studied the combination of spirulina (SM) and *Bacillus* pseudomonas (BP) to establish an alga-bacterial symbiosis (ABS) system in cement mortar, in order to enhance the efficacy of MICP after pyrolysis. The results show that adding SM alone has almost no self-healing ability under natural light conditions, while the photosynthesis of SM can act as an in situ O_2_ producer in aerobic blood pressure metabolism, thereby significantly stimulating the self-healing process. The healing rates of NL-10SM10BP increased by 21.7%, 31.5% and 13.1%, respectively, on 3, 14 and 28 days. AL-10SM10BP reached 89.8% in 3 days, while AL-10SM reached 100% in 14 days.

Despite being largely confined to laboratory-scale research and confronting hurdles in efficiency, light dependence, and long-term stability, this technology nevertheless presents a highly attractive direction for building more sustainable and resilient future infrastructure.

### 2.5. Microcapsules Remediation Technology

Microcapsules are micron-sized particles consisting of a stable outer shell encapsulating cargo, which can be solid, liquid, or gas, with wide applications in various fields. Since White et al. [75] introduced microencapsulation technology for polymer self-healing in 2001, microencapsulated healing agents have attracted extensive attention for autonomous self-healing applications. Embedding microcapsules in materials enables localized response to damage upon rupture, followed by release and activation of healing agents. The basic principle of achieving autonomous self-healing through microencapsulation is that when cracks propagate in the cementitious matrix, they mechanically rupture dispersed microcapsules, releasing their contents into the crack volume. In the case of self-healing concrete, ideally, capsules that can be easily mixed into concrete and release healing agents upon cracking are required [6].

**Figure 8:**
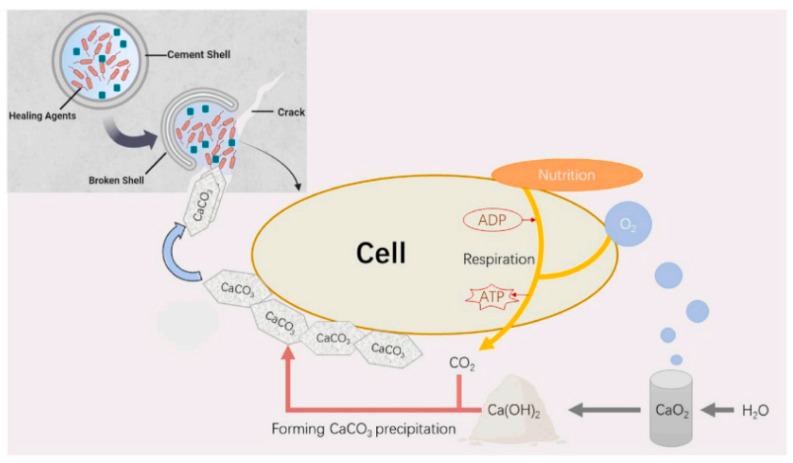
Schematic diagram of calcium oxide enhancing bacteria-induced calcium carbonate deposition after microcapsule rupture (taking Pasteurella as an example) [76].

Muhammad Arslan Ahmad et al. [76] developed an innovative approach combining oxygen self-supply strategy with strain B6 (bacteria isolated from mangrove sediments). Therefore, B6 spores were embedded in cement-shelled microcapsules along with Ca(OH)_2_ and CaO_2_ as oxygen sources and essential nutrients, constructing a microbial ecological repair system (Figure 8). Results showed successful crack repair in the B6 group (BPN) with oxygen sources and nutrients, achieving approximately 100% repair efficiency after 30 days. Jose Milla [77] et al. studied the crack repair efficiency of calcium nitrate microcapsules in steel fiber reinforced concrete beams, evaluating variables including microcapsule size, concentration (by cement weight), and capsule shell properties. Process parameters for microcapsule preparation were adjusted to control shell properties and average particle size. Liu and Fang [6] designed and prepared a novel microcapsule self-healing material promising for concrete crack repair in marine environments. With incorporation of hydration products, CSA and water addition resulted in significant repair of initial cracks. The degree and rate of self-healing process largely depended on water transport from surface to cementitious material matrix. Based on crack volume changes, repair efficiency could reach 82.60%. Significant potential in capsule-type microbial healing agents for concrete. Compared with liquid healing agents, it offers greater convenience in production, transportation, storage, and application [78]. These production and construction experiences provide valuable references for the commercialization of microbial self-healing concrete.

The large-scale and standardized production cost of microcapsules is still relatively high at present [23]. For instance, the cost of some biopolymers (such as PLA) is approximately 20% higher than that of traditional chemical materials (such as SBR) [79]. It is necessary to develop economically efficient large-scale production processes to reduce costs. Adding a large amount of capsules may affect the workability of concrete (such as fluidity and castability) and the final mechanical properties [80]. It is crucial to find the best balance point between the dosage of capsules and performance improvement. The current repair capabilities mainly target cracks at the micrometer level. For wider structural cracks, the repair effect may be limited and is usually a one-time repair, making it difficult to deal with repeated cracking at the same location [81]. Despite the challenges associated with durability and cost, microcapsule-based self-healing concrete technology continues to advance through innovations in composite wall material design and precise control of release mechanisms. Future research will concentrate on developing more robust and environmentally stable capsule systems, as well as optimizing large-scale manufacturing processes and enhancing cost-efficiency.

The microbial capsule system enables targeted repair, making it particularly suitable for key projects and emergent scenarios where repair effectiveness is critical and cost is a secondary concern. The healing process results from a synergistic effect between microorganisms and a combination of organic and inorganic additives. While the primary function of these additives is to protect the encapsulated microorganisms, certain specialized materials can also act as catalysts to enhance the repair reaction. To evaluate the efficacy of different systems, Table 3 summarizes and compares various parameters, including carrier type, microbial survival rate, healing time, and mechanical recovery.

### 2.6. Collaborative Remediation Technology of Inorganic Minerals and Microorganisms

The activity of microorganisms is governed by irreversible chemical/physical processes [82,83], The harsh conditions in concrete, such as high pressure, high temperature, alkaline environments, and insufficient oxygen availability [84], adversely affect microbial activity and viability [85]. Therefore, combining inorganic materials with microorganisms has been proposed to protect against extreme environments and harsh manufacturing processes. Current research has identified common carrier materials, including hydrogels, lightweight aggregates, PVA fibers, etc. These carrier materials can provide protection for microorganisms and release nutrients in cracks to promote self-healing [86]. During the initial hardening stage of concrete and when it is subjected to loads, uniformly distributed inorganic materials can effectively inhibit the formation of plastic shrinkage and drying shrinkage cracks [87]. Modifying inorganic materials is the main means to improve their dispersion uniformity. For example, Yang and Hu [88] discovered that the dispersion of silica can be improved greatly by catalyzing with alkali. Meanwhile, the interface transition zone between inorganic materials and the cement matrix is naturally a relatively weak and porous area. These tiny pores and cracks can precisely provide hiding places for the built-in microbial spores and their nutrient sources, forming a natural “microbial warehouse” [89]. Synergistic blocking and strengthening: Calcium carbonate crystals induced by microorganisms interweave with inorganic materials. Inorganic materials provide nucleation sites for the growth of microorganisms, while microorganisms wrap and anchor the inorganic materials, enhancing the adhesion between the inorganic materials and the substrate. This “organic-inorganic” composite repair can more effectively seal cracks and restore the mechanical properties of the structure.

**Figure 9:**
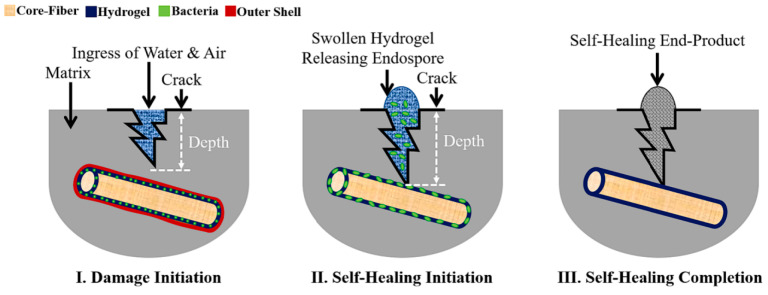
Mechanism of BioFiber-mediated concrete self-repair [90].

Mohammad Houshmand Khaneghahi et al. [90] developed a multifunctional polymer fiber (referred to as BioFiber, Figure 9) to deliver biological self-healing agents into cementitious materials. Each BioFiber is capable of producing 40–80 mg of calcium carbonate within the first 30 h of activation. Lei V. Zhang and Moncef L. Nehdi [89] investigated the potential of PVA fibers immobilized with *Bacillus* Pasteurian as a bio-green healing agent for concrete cracks. Complete healing was achieved for crack widths up to 111 μm within 90 days. SEM-EDS combined with Raman spectroscopy analysis indicated that calcite was the primary self-healing product. Nafeesa Shaheen and Rao Arsalan Khushnood [7] studied direct immobilization of *Bacillus* subtilis, evaluating two media: iron oxide nano/microparticles (INMPs) and bentonite nano/microparticles (BNMPs). Wu and Hu [91] prepared a green inorganic cementitious material using calcium carbide slag, fly ash, and desulfurized gypsum as raw materials, and coated bacterial spores with the prepared cementitious material. Germinated bacterial spores exhibited metabolic activity, producing urease which catalyzed urea decomposition and generated mineralized products that healed concrete cracks (Figure 8). Xiao and Cise Unluer [87] proposed a novel single bacterial spore capsule through layer-by-layer self-assembly of poly dimethyl diallyl ammonium chloride and silica nanoparticles to improve healing consistency and minimize negative impacts on the mechanical properties of the resulting concrete.

In summary, combining inorganic materials with microorganisms not only greatly enhances microbial activity and improves remediation efficiency, but also provides new application pathways for inorganic materials (Table 3). The synergistic interaction between organic and inorganic components not only enhances the damage repair rate but also contributes to a more balanced recovery of both strength and toughness. Nevertheless, current limitations include insufficient long-term performance data and the high cost associated with complex fabrication processes for carriers. Future research should prioritize the development of more cost-effective and scalable composite carrier systems, as well as further investigate the long-term durability and repair efficacy of these materials in real-world applications.

This article provides a systematic comparison of the aforementioned repair methods, evaluating them across five key dimensions: repair mechanism, efficiency/capability, principal advantages, main limitations, and potential application scenarios, as summarized in Table 4. Furthermore, in light of the significant differences observed in concrete cracks at various stages, this work correspondingly proposes optimal repair strategies and maintenance protocols tailored to each specific phase. The underlying repair mechanisms for these stage-specific approaches are also explicated in detail, as presented in Table 5.

## 3. Conclusions and Future Prospects

### 3.1. Conclusions

This study provides a comprehensive review of recent advancements in self-healing concrete technologies, encompassing fungal, bacterial, mixed fungal, enzyme-mediated, microcapsule, microalgae, and inorganic approaches. It underscores the diversity and innovation within this research domain. Future efforts are anticipated to focus on the synergistic integration of these methods to develop more efficient and durable self-healing concrete systems.

Under the synergistic action of fungi, bacteria, or multiple microorganisms, both the interior and surface of concrete cracks can be repaired simultaneously. These microorganisms are capable of providing continuous repair throughout a significant portion, or even the entire service life of concrete. They can heal cracks up to 2.0 mm wide, leading to a strength recovery of 20–35%. More importantly, this ongoing microbial activity mitigates the long-term strength degradation of concrete, thereby extending the service life of structures. In parallel, microalgae-based restoration enables self-healing under natural light conditions. Hybrid microbial systems and microalgae-assisted therapy represent cutting-edge research directions in this field. By mimicking natural symbiotic systems, they aim to create self-sustaining maintenance mechanisms, which hold significant potential for reducing long-term maintenance costs and advancing low-carbon building technologies. However, most of these approaches remain at the laboratory research stage.

Microcapsule embedding technology is one of the most promising implementation paths at present. It pre-protects the repair agent and only starts to repair when cracks appear, achieving true “active repair”. A key balance point lies in the fact that the capsule must be both “strong” enough to remain in the concrete for a long time and “fragile” enough to break and release the repair agent in time when cracks appear. In addition, the degradation of capsules may also lead to the formation of micro-pores inside the concrete. If not designed properly, it may have a negative impact on the long-term strength and durability of concrete.

In practical waterproofing and seepage prevention projects, notably for the maintenance and reinforcement of existing structures, organic-inorganic composite materials (e.g., water-based permeable crystalline agents) have demonstrated considerable technical and economic advantages. Their ability to utilize the inherent components of concrete to achieve long-term waterproofing and self-healing, coupled with relatively straightforward application, makes them highly applicable. However, challenges remain: the cost of multifunctional fibers and microbial preparations is still high, and there is a current lack of unified design standards and application specifications to guide engineering practice. Furthermore, quantifying the specific contribution of fibers and microorganisms to the overall repair effect requires further in-depth investigation.

### 3.2. Outlook

(1) Select and optimize microbial bacteria to enhance fracture healing in concrete. This requires identifying microorganisms that exhibit high survival and growth rates in the concrete environment, enabling the development of tailored formulations to improve healing.

(2) How to precisely control the release rate of the repair agent, the activation timing of microorganisms, and ensure that the repair material can function promptly and effectively when cracks appear remains a subject that requires meticulous design and optimization. Introducing excessive organic components or new carriers into substrates such as concrete may have unknown effects on the workability, early strength, durability, etc., of the substrate, and a comprehensive assessment is required.

(3) The majority of tests are restricted to examining small-volume materials, and there are no standardized assessment criteria for evaluating self-healing efficacy. Additionally, it remains unclear how to develop a unified evaluation standard or whether microorganisms can survive throughout the building’s service life.

(4) In future research, more theoretical, simulation and experimental studies are needed to balance the improvement of crack repair capacity and the maintenance of mechanical properties of concrete. In addition, construction in complex natural environments such as extreme cold, oxygen deficiency, high pressure and high temperature have become a trend of social development. Therefore, the adaptation of MICP technology to different natural environments and the development of corresponding types of microbial concrete or healing agents are research topics worthy of exploration.

## Figures and Tables

**Table 1:** Current Methods for Concrete Repair.

Fixes	The Main Application Is the Fracture Type	Main Construction Steps	Merit	Demerits
Apply epoxy paint	Fine and shallow cracks that are difficult to fill in the slurry, cracks that do not stretch, and cracks that are no longer active.	(1) Clean and dry the surface near the crack. (2) Fill with epoxy cement, and evenly apply the cement to scrape the surface of the fracture.	Improve the stain resistance of concrete, increase the strength of concrete, and prolong the service life of concrete.	The difficulty of construction is increased (a certain proportion needs to be mastered, and there are certain requirements for the experience and skills of construction personnel.)
Filling method	Patch wider cracks, shallow deep cracks.	The grouting material used for repairing cracks is pressed into the crack cavity at a certain pressure within a certain period of time.	The loss rate is small, which helps to improve the recovery rate of concrete resources and has strong adaptability.	The process is complex, which increases the difficulty and time of operation, the cost is high, and the requirements for filling materials are high.
Secondary dough pressing method	This crack is usually found on the surface of a newly poured structural component that is exposed to air, and this crack is neither deep nor wide.	(1) The plastic concrete adopts the method of pressing and wiping once. (2) The hardened concrete infiltrates the cement slurry into the crack.	Improve the flatness and solidity of the concrete surface, enhance the compactness and durability of concrete, and improve the frost resistance and weathering resistance of concrete.	Long repair time and high repair requirements; Avoid plastering under the scorching sun or direct sunlight to avoid premature drying and cracking of concrete; It may not be cured in place, which will affect the strength of concrete
Surface strip method	For cracks, the range of movement is not limited to one plane, and there are waterproof requirements, and it is inconvenient to chisel and repair the active cracks.	(1) Place a flexible rubber sealing strip on top of the crack. (2) Use a binder to bond the periphery to the concrete, so that the sealing strip can move freely with the cracks.	The bond is firm, not easy to fall off, excellent performance, has a strong “self-repair” function, good durability and easy construction, and there is no material waste in the seam tape.	The cracks are clean, the cracks are completely dry, and the construction temperature is suitable.

**Table 2:** Bacteria vs. Fungi: Comparison Table of Concrete Repair Performance.

Performance Indicators	Bacteria (Taking *Bacillus* Pasteurella and Other Urease Bacteria as Examples)	Fungi (Taking Various Filamentous Fungi as Examples)
Main repair mechanism	Biomineralization: Through metabolic activities (such as urea hydrolysis), calcium carbonate (CaCO_3_) crystals are induced to form, achieving chemical bonding and healing.	Physical filling: Mechanically filling and lapping cracks through a rapidly growing mycelial network.
Crack healing width	It is relatively wide and can usually effectively repair cracks of 0.2 to 0.5 mm, with some research reports reaching 1 to 2 mm.	It is relatively narrow and mainly suitable for micro-cracks of less than 0.2 mm. The mycelium is difficult to form effective support in wider cracks.
CaCO_3_ content/deposition capacity	High. This is its core repair mechanism. The deposition rate of CaCO_3_ is directly related to the repair effect and can significantly enhance the density.	Extremely low or even none. Fungi mainly rely on physical filling and basically do not produce or only produce trace amounts of minerals with cementing effects.
pH tolerance	Strong. Especially alkali-resistant *Bacillus*, its spores can survive for a long time in the highly alkaline environment of concrete (pH > 12) and be activated when the cracks come into contact with water.	Moderate to weak. Although some fungi have a certain degree of alkali tolerance, the extremely alkaline environment inside concrete can severely inhibit the germination of their spores and the growth of mycelium.
Strength recovery (compressive strength/flexural strength	Significant. Calcium carbonate crystals chemically combine with the concrete matrix, which can effectively restore mechanical properties, and the strength recovery rate usually reaches over 90%.	Limited and uncertain. Mycelial filling can temporarily restore a certain degree of impermeability, but it may form a weak interface, making little contribution to the long-term recovery of mechanical properties. It may even have a negative impact due to the introduction of water and organic matter.
Long-term stability	High. The generated calcium carbonate is an inorganic mineral with stable properties, which can coexist perfectly with the concrete substrate and provide a long-lasting repair effect.	Low. Mycelium is an organic substance. When it dries out and its nutrients are exhausted, it will die and decompose, leaving new gaps. The repair effect may only be temporary.

**Table 3:** A comparison between microbial capsules and the combination of inorganic and organic substances.

	Microbial Capsules	The Combination of Inorganic and Organic
Core component	Shell: Urea-formaldehyde resin, gelatin, etc. Core material: Microbial spores, nutrients	Microbial spores + inorganic gel materials such as sodium alginate
Repair mechanism	Passive trigger: Crack expansion pierces the capsule, releasing the healing agent to polymerize and bond	Synergistic repair: Microorganisms produce calcium carbonate to form a framework, and calcium alginate gel instantly blocks leaks and fills voids
Typical healing rate	The initial repair efficiency is relatively high, reaching 80% to 95%, but it is usually one-time	For a 1 mm crack, the 7-day strength recovery rate is approximately 22.7%, and it can achieve instantaneous leakage sealing with the water seepage coefficient reduced from 9.76 to 0
Main advantages	The repair speed is fast and the efficiency of repairing specific cracks is high	Combining the advantages of both: the repair product is more flexible, can achieve instantaneous leak sealing, and the repair rate and effect are improved
Main challenges and limitations	1. Strength influence: As inclusions, it may reduce the initial strength of concrete 2. High cost: The preparation and mixing processes are complex 3. Single repair: Usually, it can only be repaired once 4. Trigger mechanism: The crack needs to expand large enough to puncture the capsule	1. Complex process: The preparation and quality control of multi-component composite systems have high requirements 2. Research stage: Mostly laboratory research, long-term performance and engineering application data need to be accumulated

**Table 4:** The core repair mechanisms, repair efficiency/capabilities, main advantages, main limitations, and potential application scenarios of different repair methods.

Repair Technology	Repair Mechanism	Repair Efficiency/Capability	Main Advantages	Main Limitations	Potential Application Scenarios
Fungal repair	Physical filling of the mycelial network may be accompanied by biomineralization	It can form a dense mycelial network; The mineralization ability is usually lower than that of bacteria	The mycelial network has strong expansion capabilities and may achieve long-distance repair	The survival challenge is significant: The survival rate is low in the highly alkaline environment of concrete	It is still in the exploratory stage and may be used for surface sealing where strength requirements are not high
Bacterial repair	Microbial mineralization: Metabolism generates calcium carbonate (CaCO_3_) precipitate to seal the cracks	It can repair micro-crackers smaller than 0.2 mm. Field tests have reduced the capillary water absorption coefficient of the concrete surface by over 90%	Technology is relatively mature, the restoration effect is clear, and the mineral sedimentation is stable and long-lasting	It is necessary to ensure the survival of bacteria in concrete. Common bacterial strains may require external nutrition	Surface repair and micro-crack repair: It is suitable for treating surface defects and existing micro-cracks in concrete
Mixed microbial system	Different microorganisms (such as fungi and cyanobacteria) work together to complete mineralization	Under the synergy, microorganisms are healthier and more efficient, and can form more calcium carbonate	With complementary functions, it is expected to achieve self-nutrition supply and reduce reliance on external substances	The balance and control of microbiota are difficult, the system is complex, and the research is still in its early stage	A fully autonomous and self-repairing system for the future, inspired by the lichen symbiotic system
Enzyme-induced calcium carbonate precipitation (EICP)	Directly utilize urease to catalyze the hydrolysis of urea and induce calcium carbonate precipitation	Quick and direct response, high efficiency; Omit the microbial culture process	The process is simplified, and the cost is relatively low (such as extracting urease from soybeans); There is no need to maintain microbial activity	Enzymes may become inactive in a concrete environment. One repair, lacking the ability to sustain repairs	Soil reinforcement and dust control: They also have application prospects in crack repair
Microcapsule encapsulation	Repair agents (such as bacteria, nutrients, enzymes) are embedded in microcapsules and released when the cracks expand	The 28-day compressive strength recovery rate of mortar specimens mixed with 1% microcapsules can reach 80%	Precise positioning, the repair agent is well protected, which can significantly increase the survival rate of microorganisms in concrete	The interface bonding with the cement matrix is a key challenge; It is usually a one-time repair	Built-in self-healing concrete: Suitable for critical structures that have self-healing requirements for early micro-cracks
Microalgae assist in healing	Carbon and nitrogen are obtained from the air by nitrogen-fixing cyanobacteria (such as anabaena and Candida), and they work together with fungi to produce calcium carbonate	It grows well in a laboratory environment with only air and light and can form calcium carbonate	Theoretically, it can be fully autotrophic, requiring only air, sunlight and water, without the need for additional nutrients	Heavily dependent on light, its application in underground or indoor structures is limited; The research is very preliminary	The conceptual self-sustaining repair system in extreme environments is currently only a laboratory concept
Organic-inorganic mixed repair	Inorganic active substances penetrate with water as the carrier, catalyzing the further hydration of unhydrated cement forming crystals and seal the pores	The reaction speed is fast, and the leakage can be solved within 3 days. The maintenance cost can be reduced by 70% to 90%	Good compatibility with concrete; Long-lasting performance (dormant when dry, reactivated when exposed to water)	The repair relies on unhydrated cement particles, and the effect on aged concrete or repeated repairs may diminish	Waterproofing and anti-seepage maintenance for new construction projects and anti-seepage and leakage repair for existing structures

**Table 5:** A comparison of damage between newly built structures and aging buildings.

	Early Micro-Cracks in Newly Built Concrete	Damage to Long-Term Aging Structures
Repair strategy	“Proactive preventive” self-repair	“Passive intervention-based” repair and enhancement
Objective	It automatically repairs immediately when cracks appear (usually less than 0.5 mm) to prevent their expansion and enhance durability.	Repair the already formed wide cracks (possibly >0.5 mm) to restore the integrity and load-bearing capacity of the structure.
Recommended microbial types	Alkali-resistant *Bacillus*, spore-forming bacteria	Urease bacteria, complex microbial communities, and strains with strong environmental adaptability
Repair method	It is directly added to the concrete mixture during the production process.	When repairing, apply it to the cracked area by injection or spraying.
Microbial protection strategy	Microcapsule encapsulation, porous aggregate loading, hydrogel encapsulation.	It does not require long-term protection and is usually prepared in a repair slurry containing nutrients for direct use.
Nutrient source	It is encapsulated together with microorganisms or evenly dispersed in the concrete matrix.	It is included in the repair slurry or gel and injected into the crack along with the bacterial liquid.
Mechanism of action	The pre-buried microorganisms are activated when water enters the cracks, generating CaCO_3_ to seal the cracks.	Under the suitable environment and nutrients provided by humans, microorganisms rapidly mineralize inside the cracks.
Key advantages	Fully automatic, timely and requiring no manual intervention, it is particularly suitable for hard-to-reach areas.	It has high flexibility and can “prescribe the right medicine” based on the specific condition of the injury. The selection of bacterial strains is not subject to the long-term limitation of the initial high alkalinity of concrete.
Main challenges	1. Ensure the long-term survival rate of microorganisms throughout the decades-long lifespan of concrete. 2. The repair capacity for wider cracks is limited. 3. It may have a slight impact on initial workability and strength.	1. Manual operation is required, and accessibility is the prerequisite. 2. Uniformity and depth control of the repair effect. 3. The repair cost is usually higher than that of preventive incorporation.

## Data Availability

No new data were created or analyzed in this study. Data sharing is not applicable to this article.
